# Therapeutic scarification, shadow pain, and integrative geriatric rehabilitation for chronic musculoskeletal pain in older adults in Cameroon: a prospective mixed-methods observational study

**DOI:** 10.3389/fpain.2026.1826721

**Published:** 2026-06-08

**Authors:** Ibrahim Npochinto Moumeni, Abdel-Nasser Njikam Moumeni, Jean-Marie Alima, France Mourey, Faustin Atemkeng Tsatedem

**Affiliations:** 1 Department of Physiotherapy & Physical Medicine, Faculty of Medicine and Pharmaceutical Sciences, University of Dschang, Dschang, West Region, Cameroon; 2 Department of Physical Medicine & Osteopathy, Regional Hospital of Bafoussam, Bafoussam, West Region, Cameroon; 3 Institute for Applied Neurosciences and Functional Rehabilitation (INAREF), Odza-Yaoundé, Cameroon; 4 Franco-African Center for Applied Rehabilitation and Health Sciences (CFARASS), Foumbot, West Region, Cameroon; 5 Department of Geriatrics and Gerontology, Sorbonne Université, Pitié-Salpêtrière Hospital, Paris, France; 6 Licensed Physiotherapy Practitioner, Vitry-sur-Seine, Île-de-France, France; 7 Lecturer in Neurorehabilitation, Faculty of Health Sciences, University of Parakou, Parakou, Benin; 8 Secretary General, French-Speaking African Society for Neurorehabilitation (SAFNeR), Parakou, Benin; 9 UREKIM—Research Unit in Physiotherapy and Physical Medicine, University of Dschang, Dschang, West Region, Cameroon; 10 CRESHDEM, Faculty of Medicine and Pharmaceutical Sciences, University of Dschang, Dschang, West Region, Cameroon; 11 INSERM UMR1093 CAPS, University of Burgundy, Dijon, France

**Keywords:** chronic musculoskeletal pain, culturally adapted care, fall risk, geriatric complications, integrative gerontological rehabilitation, mixed-methods, older adults, proprioceptive alterations

## Abstract

**Background:**

In sub-Saharan Africa, the coexistence of conventional medicine and traditional healing practices creates a complex therapeutic landscape that disproportionately affects older adults. Therapeutic scarification—cutaneous incisions performed on painful body areas—remains one of the most widespread traditional approaches to chronic pain management. Its geriatric-specific complications and functional consequences remain poorly documented. With a projected 67% increase in the population aged over 60 in Cameroon by 2030 and fewer than 15% of older adults benefiting from effective social coverage, this represents a major public health priority.

**Objective:**

To analyze the prevalence, typology, and geriatric-specific complications of therapeutic scarification among older adults with chronic musculoskeletal pain in Cameroon, and to evaluate the preliminary effectiveness of a culturally integrative physiotherapy approach bridging traditional and conventional medicine.

**Methods:**

A prospective single-center mixed-methods observational study was conducted from February 2023 to May 2025 at Bafoussam Regional Hospital. Patients aged ≥65 years with chronic articular pain (≥6 months) and documented history of therapeutic scarification were included. The quantitative component involved standardized clinical assessment and statistical analysis; the qualitative component comprised semi-structured interviews (*n* = 92, 30–45 min) analyzed thematically following Braun and Clarke's framework (kappa = 0.78). A culturally adapted integrative physiotherapy protocol over 16 sessions was implemented, with follow-up of 6–18 months.

**Results:**

Ninety-two patients were enrolled (mean age 73.1 ± 8.4 years; 60 women, 65.2%). Four scarification modalities were identified: parallel linear (63.0%), punctiform (20.7%), deep cruciform (12.0%), micro-scarification with cupping (4.3%). Knee osteoarthritis predominated (44.6%). Complications included infections (15.2%), keloids (34.8%), and proprioceptive impairment (35.9%) associated with a 29% increase in relative fall risk (*p* < 0.01). A significant age-complication gradient was observed (OR = 4.9 for >75 vs. 65–70 years; *p* = 0.032). Education level was inversely correlated with scarification frequency (*r* = −0.67; *p* < 0.001). Among 68 patients receiving the integrative approach, 73.5% reduced scarification use (*p* < 0.001), with significant functional improvements and 89.7% patient satisfaction. Five qualitative themes of adherence drivers were identified.

**Conclusion:**

Therapeutic scarification exposes older adults to preventable geriatric complications, including a previously unrecognized fall risk from proprioceptive disruption. The “shadow pain” mechanism—transient nociceptive inhibition by iatrogenic pain—offers a novel neurophysiological explanation for treatment adherence. The culturally integrative rehabilitation model achieved preliminary effectiveness supporting development of context-sensitive geriatric care strategies in low-resource settings. Randomized controlled trials are needed to confirm these findings.

## 1 Introduction

In sub-Saharan Africa, the coexistence of conventional biomedicine and traditional healing practices defines a complex therapeutic landscape that particularly affects older adults (1, 2). In Cameroon, this reality is amplified by progressive demographic aging, with an estimated 67% increase in the population aged over 60 projected by 2030, occurring in a context where fewer than 15% of older adults benefit from effective social coverage (3–9). This demographic transition, occurring alongside persistent health system fragilities, creates a situation where traditional practices frequently serve as primary or complementary healthcare for a rapidly growing elderly population.

Therapeutic scarifications—locally designated as “ndongsè” in Bamiléké, “ntouba” in Ewondo, “mbako” among the Bamoun—constitute one of the most widespread traditional practices for managing chronic pain in West and Central Africa (4). These interventions consist of cutaneous incisions, superficial to moderately deep, performed on painful body areas with the aim of “extracting” pain conceptualized as a mobile entity within local belief systems (5, 10–16). The practice is deeply embedded in cultural representations of the body, illness, and aging.

Our clinical experience at Bafoussam Regional Hospital, spanning over a decade of geriatric musculoskeletal care, reveals that approximately 90% of patients aged over 65 years who consult for chronic articular pain have previously undergone therapeutic scarification (17–21). This high prevalence is driven by multiple factors: perceived failure or inaccessibility of conventional medicine, economic and geographic barriers to specialist care, intergenerational transmission of traditional practices, and a phenomenon we have conceptualized as “shadow pain” (*douleur-ombre*)—a transient nociceptive inhibition whereby the iatrogenic pain of scarification temporarily masks the underlying chronic pain (6, 11, 22–24).

Although persistence of scarification practices is often interpreted as purely cultural, our observations suggest a specific neurophysiological mechanism. Shadow pain, defined as transient masking of initial chronic pain by iatrogenic nociceptive stimulation induced by cutaneous injury, is consistent with well-established counter-irritation and descending inhibitory control pathways, in which peripheral nociceptive input temporarily suppresses pre-existing pain signals (25–30). In older adults, age-related alterations in central pain modulation may prolong this masking effect and increase perceived scarification efficacy.

The Cameroonian legislative framework adds complexity: Presidential Decree No. 2007/0548 of April 5, 2007, recognizing traditional medicine as a legitimate therapeutic alternative, creates a paradoxical situation where practices carrying significant risks for elderly populations benefit from official state recognition (7, 31–33). This context, combined with the near-absence of rehabilitation medicine from medical education curricula in Central Africa (8, 9), means older adults often navigate between two therapeutic systems without professional guidance. The systematic therapeutic nihilism documented among healthcare professionals across Central Africa further reinforces recourse to traditional practitioners (34).

While the anthropological dimensions of traditional African medicine have been extensively documented (1, 4, 5, 10, 35), the specific geriatric complications of scarification—including impact on proprioception and fall risk—remain virtually unexplored. Although the WHO advocates for respectful integration of traditional and conventional medicines (11, 12, 36–38), concrete models for geriatric musculoskeletal care are lacking.

**Figure 1:**
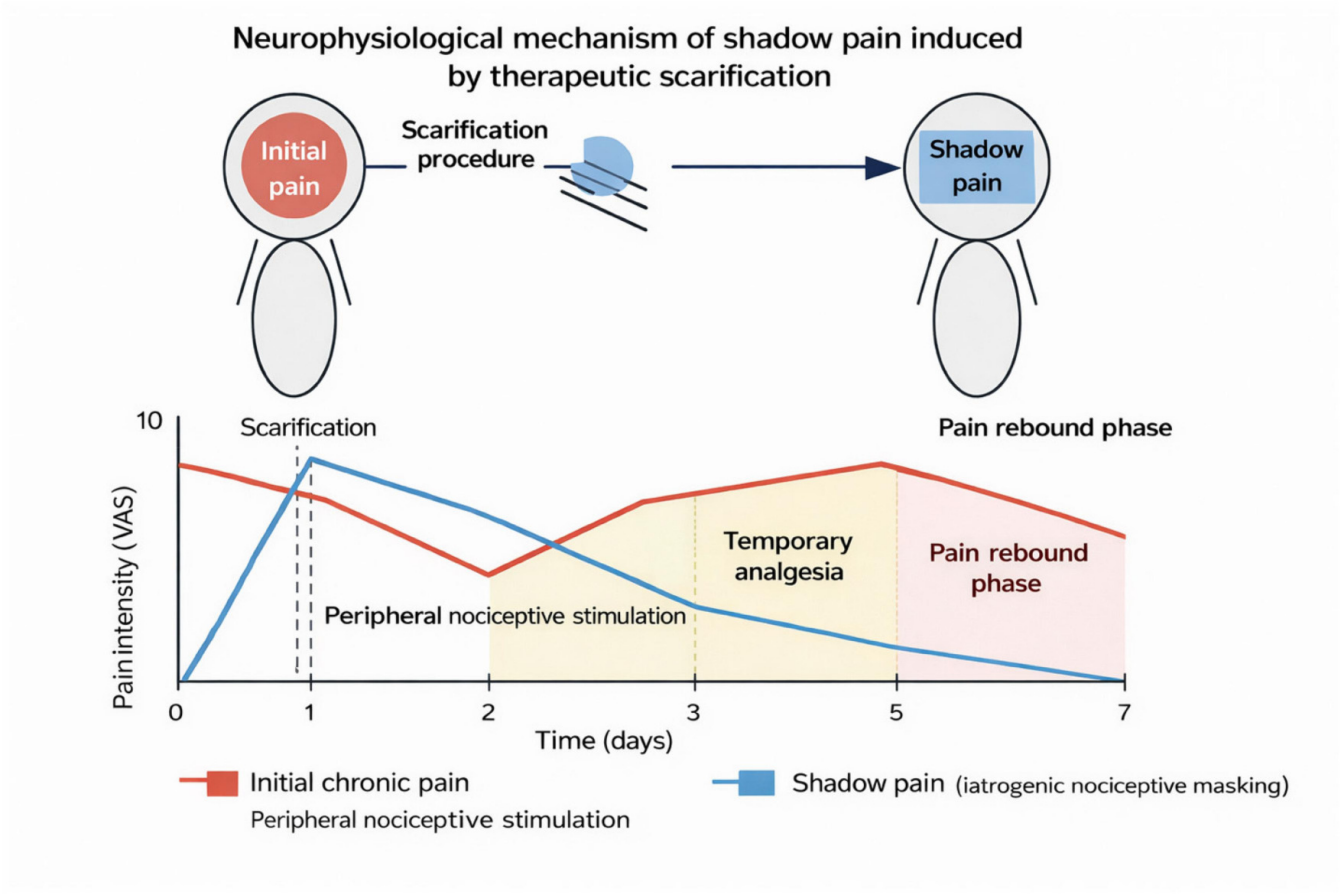
Shadow pain mechanism: transient nociceptive masking induced by therapeutic scarification. Schematic representation illustrating the temporary inhibition of initial chronic pain (red curve) by iatrogenic nociceptive stimulation from scarification (blue curve), mediated by counter-irritation and activation of descending inhibitory pathways, leading to transient analgesia of approximately 3–5 days. Perceived relief reinforces adherence to traditional practices. Pain rebound is more pronounced in older adults (+31%), creating an amplified dependency cycle.

**Figure 2:**
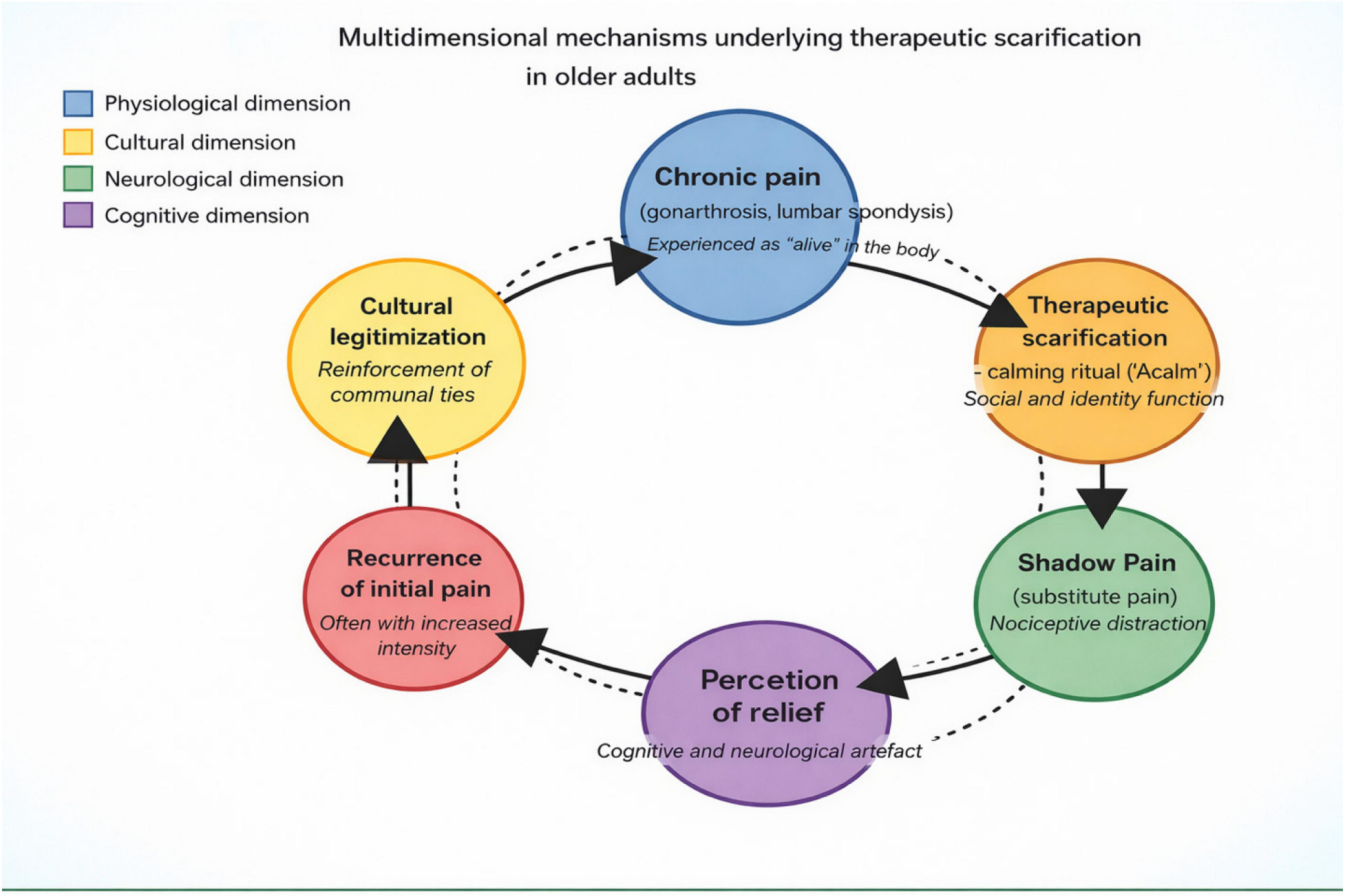
Multidimensional cultural and neuro-sensory cycle underlying therapeutic scarification adherence in older adults. Conceptual diagram illustrating the dynamic interaction between physiological, neurological, cultural, and cognitive dimensions in the persistence of scarification practices. The cycle begins with chronic musculoskeletal pain, followed by therapeutic scarification as a culturally legitimized ritual. Shadow pain induces transient analgesia, reinforcing cultural beliefs and neurocognitive pain modulation. Pain recurrence and amplification complete the loop, which is interrupted at multiple points by the integrative protocol.

This study aimed to: (1) systematically analyze scarification practices and their geriatric-specific complications in older adults in western Cameroon; and (2) evaluate the preliminary effectiveness of an integrative gerontological model grounded in cultural competence and evidence-based rehabilitation. The shadow pain mechanism and the multidimensional cultural cycle are illustrated in Figures 1, 2.

## 2 Materials and methods

### 2.1 Study design and setting

This prospective mixed-methods observational descriptive single-center study was conducted from February 2023 to May 2025 at the Department of Physical Medicine and Rehabilitation, Bafoussam Regional Hospital, Cameroon. The mixed-methods design was driven by dual objectives: quantifying the clinical consequences of scarification (quantitative strand) and understanding the cultural dimensions of adherence (qualitative strand). These were conducted concurrently and integrated at the level of data interpretation, following the convergent parallel design described by Creswell and Plano Clark.

### 2.2 Ethical considerations

This evaluation received institutional ethical certification (No. 43/DRSO/HRB/55/2023) from Bafoussam Regional Hospital, conducted in accordance with the Declaration of Helsinki. Written informed consent was obtained from all participants, with systematic anonymization and secure data storage. Written consent was obtained specifically for photographic documentation.

### 2.3 Participants

Inclusion criteria: (a) age≥65 years—consistent with the WHO Africa-specific definition of older adults in low-resource settings, where functional aging thresholds justify this lower bound compared to the ≥60 threshold used in some settings; (b) chronic articular pain ≥6 months; (c) documented history of at least one therapeutic scarification session; (d) written informed consent.

Exclusion criteria: (a) acute pathologies (<4 weeks' duration); (b) cognitive impairment limiting meaningful communication and consent, assessed by MMSE (<18). The initial criterion “refusal to participate” has been removed from the exclusion list, as it is inherent to the consent process and not an exclusion criterion *per se*.

### 2.4 Data collection

Data collection comprised five complementary approaches. First, anthropometric and sociodemographic data were recorded. Second, semi-structured interviews of 30–45 min were conducted using a guide developed through three pilot interviews and covering six domains: (1) history of scarification use; (2) motivations and expectations; (3) perceived efficacy and side effects; (4) family and social dynamics; (5) prior experience with conventional medicine; (6) openness to alternatives. Interviews were audio-recorded, transcribed verbatim, and translated into French where necessary. Thematic analysis followed Braun and Clarke's six-phase framework. Two independent coders analyzed a 30% random subsample (kappa = 0.78, substantial agreement). Data saturation was reached at approximately the 68th interview.

Third, standardized clinical examination included: pain intensity by Visual Analog Scale (VAS) (39); functional status by WOMAC index for knee osteoarthritis (40) and Oswestry Disability Index for low back pain (41); radiological grading by Kellgren-Lawrence classification (42); and detailed scar analysis.

Fourth, proprioceptive function was assessed using two standardized tests applied to both scarified and non-scarified body segments: (a) joint position sense using passive goniometric repositioning (target angle ± 5 degrees threshold); (b) single-leg standing balance test (maximum 30 s, eyes open then closed). The same examiner administered all tests throughout the study. Within-patient comparison between scarified and non-scarified segments provided an internal control.

Fifth, photographic documentation of all scarification scars was performed with written consent.

### 2.5 Integrative therapeutic protocol

Protocol development: The protocol was developed through a three-stage process. Stage 1 involved systematic ethnographic observation of traditional scarification sessions (*n* = 12) and structured interviews with seven traditional healers. Stage 2 aligned these frameworks with evidence-based rehabilitation principles, drawing on manual therapy mechanisms (13), culturally adapted education frameworks (30, 32), and neurophysiological pain modulation models (25). Stage 3 involved iterative pilot testing with the first 12 patients. The final protocol was standardized across all participants through a written manual.

**Phase 1 (Sessions 1**–**2):** Culturally adapted evaluation using local pain vocabulary; exploration of pain representations; identification of motivations.

**Phase 2 (Sessions 3**–**4):** Contextualized therapeutic education using local metaphors; introduction of the shadow pain concept; presentation of alternatives as “modern evolutions” of ancestral practices.

**Phase 3 (Sessions 5**–**12):** Dry cupping along traditional energetic pathways; adapted acupuncture; instrumental manual therapy; gentle articular mobilizations adapted to scarified elderly skin.

**Phase 4 (Sessions 13**–**16):** Self-exercises; pain recurrence management strategies; active family involvement.

Attrition management: Strategies included flexible scheduling, weekly telephone contact during Phases 1–2, bi-weekly during Phases 3–4, family involvement from Session 2, and home visits for mobility-limited patients (*n* = 8). Four deaths (5.9%) and three losses to follow-up were recorded; 61 patients completed the full protocol.

### 2.6 Statistical analysis

Statistical analysis used SPSS v28.0 and R v4.2.0. Descriptive statistics included means ± SD for continuous variables and frequencies (%) for categorical variables. Bivariate analyses employed chi-square and Student's *t*-test (*p* < 0.05). Multivariate logistic regression identified predictive factors for integrative approach success. “Integrative approach success” was operationally defined *a priori* as the simultaneous achievement of: (a) reduction of ≥1 scarification session during 6-month follow-up; (b) VAS pain decrease ≥2 points; (c) patient satisfaction ≥7/10 at final follow-up. Pearson's method was used for normally distributed correlations.

Sample size: Given the exploratory nature of this study—the first systematic investigation of scarification-related geriatric complications in this population—a formal *a priori* sample size calculation was not performed. The sample of 92 represents the total eligible population during the 27-month recruitment period (consecutive sampling). This is acknowledged as a limitation. *post-hoc* sensitivity analysis suggests a minimum of 38 per group in a future RCT (alpha = 0.05, power = 0.80) based on the observed effect size.

## 3 Results

Analysis of data from 92 patients revealed specific patterns of therapeutic scarification use and confirmed the preliminary effectiveness of the integrative approach. Results are presented sequentially: population characteristics, scarification typology, radiological-clinical paradox, complications, sociodemographic determinants, and integrative approach outcomes.

### 3.1 Population characteristics

The study enrolled 92 patients: 60 women (65.2%) and 32 men (34.8%), mean age 73.1 ± 8.4 years. Table 1 presents detailed demographic, clinical, and socioeconomic characteristics.

**Table 1:** Demographic and clinical characteristics of the study population (*n* = 92).

Variables	n (%) or Mea*n* ± SD
Sex	
Female	60 (65.2%)
Male	32 (34.8%)
Mean age (years)	73.1 ± 8.4
Age groups	
65–70 years	28 (30.4%)
71–75 years	35 (38.0%)
>75 years	29 (31.5%)
Marital status	
Married	64 (69.6%)
Widowed	14 (15.2%)
Single/Divorced	14 (15.2%)
Education level	
No formal education	32 (34.8%)
Primary	39 (42.4%)
Secondary	18 (19.6%)
Higher education	3 (3.3%)
Comorbidities	
Type 2 diabetes	18 (19.6%)
Hypertension	34 (37.0%)
Overweight/Obesity (BMI ≥ 25)	55 (59.8%)

Data expressed as *n* (%) or mean ± SD. BMI, Body Mass Index; diagnosed per WHO criteria.

### 3.2 Distribution of chronic pathologies treated by scarification

**Figure 3:**
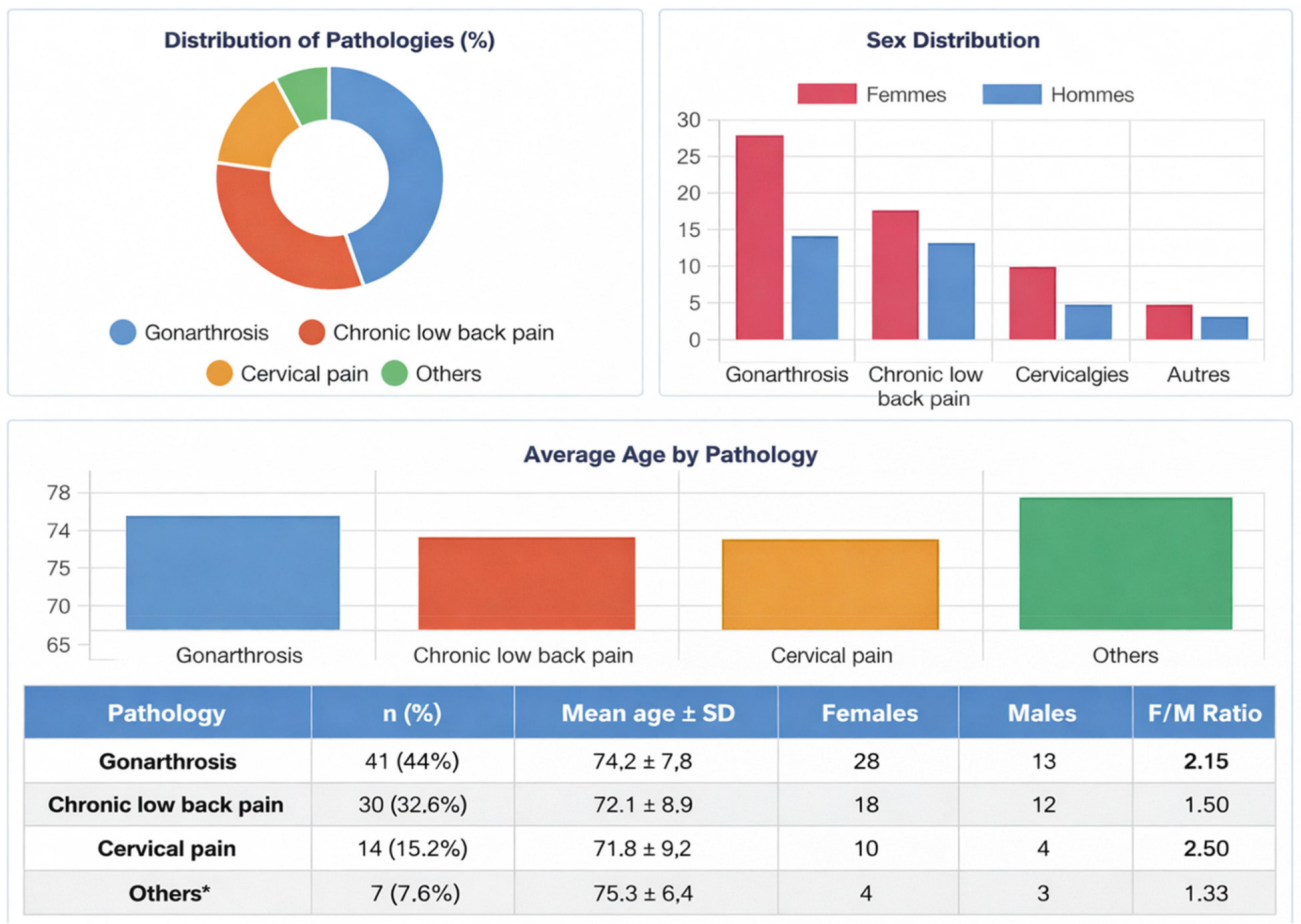
Distribution of chronic musculoskeletal conditions treated by therapeutic scarification according to age group and sex (*n* = 92). Overall percentage distribution. Age-stratified distribution across three age groups. Sex-stratified proportions. Mean age by pathology group. LBP, Low back pain. F/M, Female-to-male ratio. *Others: coxarthrosis and polyarthritis.

Knee osteoarthritis was the predominant pathology (*n* = 41, 44.6%), followed by chronic low back pain (*n* = 30, 32.6%), cervicalgia (*n* = 14, 15.2%), and others including coxarthrosis and polyarthritis (*n* = 7, 7.6%). The female-to-male ratio was highest for cervicalgia (2.50) and knee osteoarthritis (2.15). The detailed distribution is presented in Table 2. Figure 3 presents the age-stratified and sex-stratified distributions in graphical form, providing information complementary to—and not redundant with—Table 2.

**Table 2:** Distribution of chronic pathologies treated by scarification, by age and sex (*n* = 92).

Pathology	n (%)	Mean age ± SD	Women n	Men n	F/M ratio	BMI ≥ 25 (%)
Gonarthritis	41 (44.6%)	74.2 ± 7.8	28	13	2.15	32 (78.0%)
Chronic LBP	30 (32.6%)	72.1 ± 8.9	18	12	1.50	16 (53.3%)
Cervicalgia	14 (15.2%)	71.8 ± 9.2	10	4	2.50	—
Others^a^	7 (7.6%)	75.3 ± 6.4	4	3	1.33	—

a Others: coxarthrosis (*n* = 4) and polyarthritis (*n* = 3). LBP, Low back pain. F/M, Female-to-male ratio. Diagnosis by standardized clinical examination and imaging.

Figure 3 illustrates the distribution of pathologies with age-stratified breakdown across the three age groups (65–70, 71–75, >75 years) and sex-stratified proportions, providing visual information complementary to Table 2.

### 3.3 Scarification typology and ethnolinguistic classification

**Figure 4:**
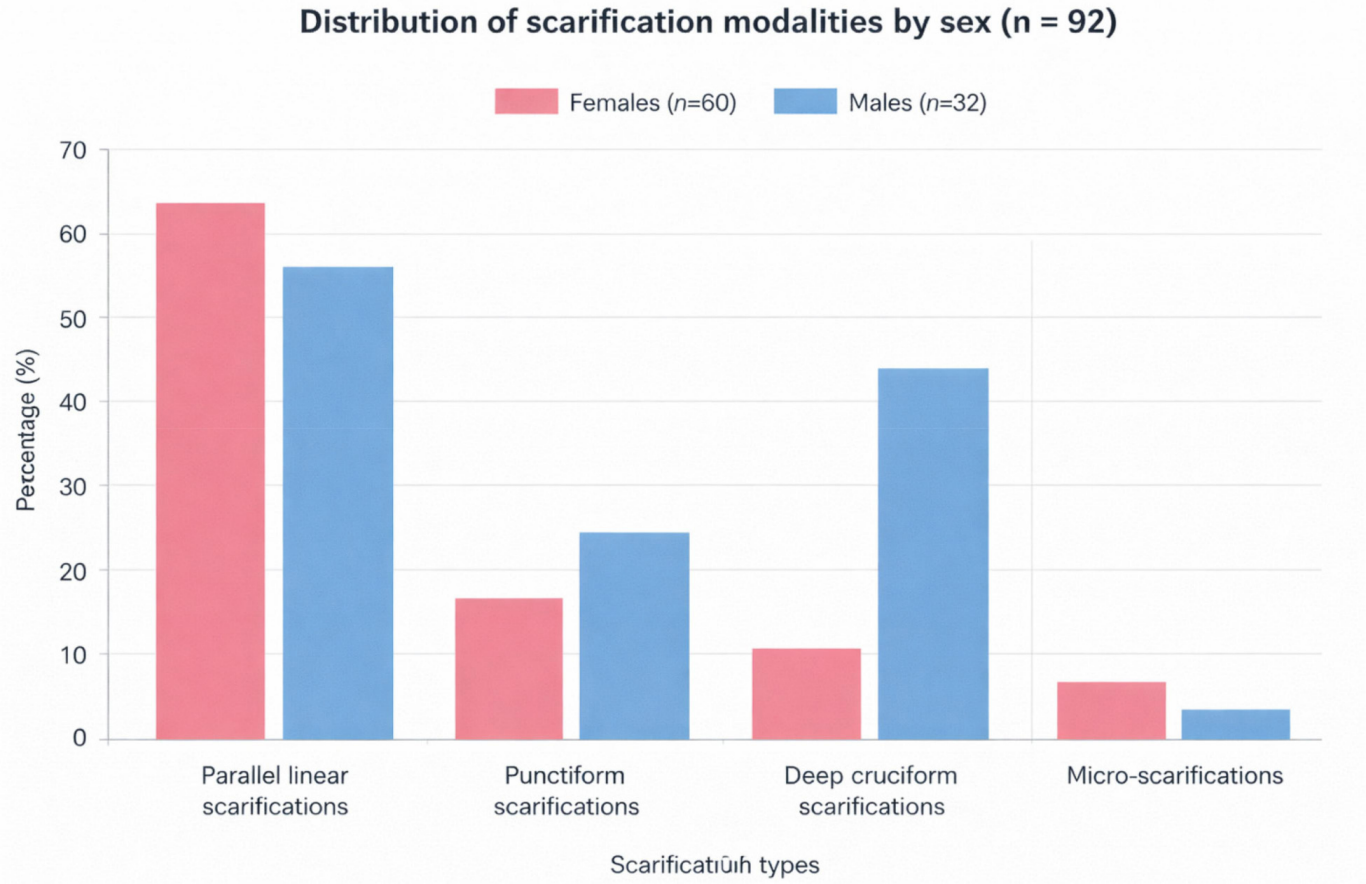
Distribution of scarification modalities according to sex (*n* = 92). Parallel linear scarifications were the most common in both sexes. Deep cruciform scarifications were predominantly observed in men (72.7%), reflecting gender-related cultural representations of pain tolerance and social status (*p* = 0.004). Micro-scarifications showed no major sex difference.

Four principal scarification modalities were identified. Their ethnolinguistic designations, prevalence, and sex distributions are presented in Tables 3, 4 (*Multivariate logistic regression)*. Figure 4 presents the sex-stratified distribution graphically.

**Table 3:** Typology of therapeutic scarifications: ethnolinguistic classification, prevalence, and sex distribution (*n* = 92).

Scarification type	Local name	n (%)	Women *n* (%)	Men *n* (%)	*p*-value
Parallel linear	Mbako	58 (63.0%)	38 (65.5%)	20 (34.5%)	0.312
Punctiform	Ndongsè	19 (20.7%)	14 (73.7%)	5 (26.3%)	0.187
Deep cruciform	Ntcheu	11 (12.0%)	3 (27.3%)	8 (72.7%)	0.004^b^
Micro + cupping	Bekop	4 (4.3%)	3 (75.0%)	1 (25.0%)	0.621

b *p* < 0.01. Chi-square test for sex distribution across types. Deep cruciform scarifications were quasi-exclusively performed on older men of high social status.

**Table 4:** (New—added in revision). Multivariate logistic regression: all variables entered, adjusted ORs, 95% CI, and *p*-values for integrative approach success (*n* = 68).

Variable	Entered in model	Adjusted OR (95% CI)	*p*-value	Significance
Formal education (primary or above)	Yes	2.15 (1.23–3.76)	0.007	**
Symptom duration <2 years	Yes	1.89 (1.12–3.18)	0.017	*
Age (per decade)	Yes	0.82 (0.64–1.05)	0.118	NS
Female sex	Yes	1.34 (0.87–2.06)	0.182	NS
Absence of comorbidities	Yes	1.67 (0.98–2.84)	0.059	NS
Scarification type (cruciform)	Yes	0.71 (0.44–1.15)	0.163	NS

(new, added in revision). All six candidate variables entered simultaneously into logistic regression. Model fit: Hosmer-Lemeshow test *p* = 0.74 (adequate fit). OR, Odds Ratio; CI, Confidence Interval; NS, Not Significant.

** *p* < 0.01.

* *p* < 0.05.

### 3.4 Radiological-clinical paradox

A notable radiological-clinical discordance was observed in 55 patients (59.8%). This paradox refers to the well-documented dissociation between structural joint damage on imaging and clinical pain severity: 26 patients (28.3%) presented Kellgren-Lawrence grades III–IV with VAS ≤ 4/10 (severe structural damage with only moderate pain), while 29 (31.5%) had grades I-II with VAS > 7/10 (mild imaging with severe pain). This discordance, well-established in the osteoarthritis literature (14, 15), contributes in our context to patient incomprehension of biomedical explanations and reinforces adherence to traditional explanatory models.

### 3.5 Geriatric-specific complications

**Figure 5:**
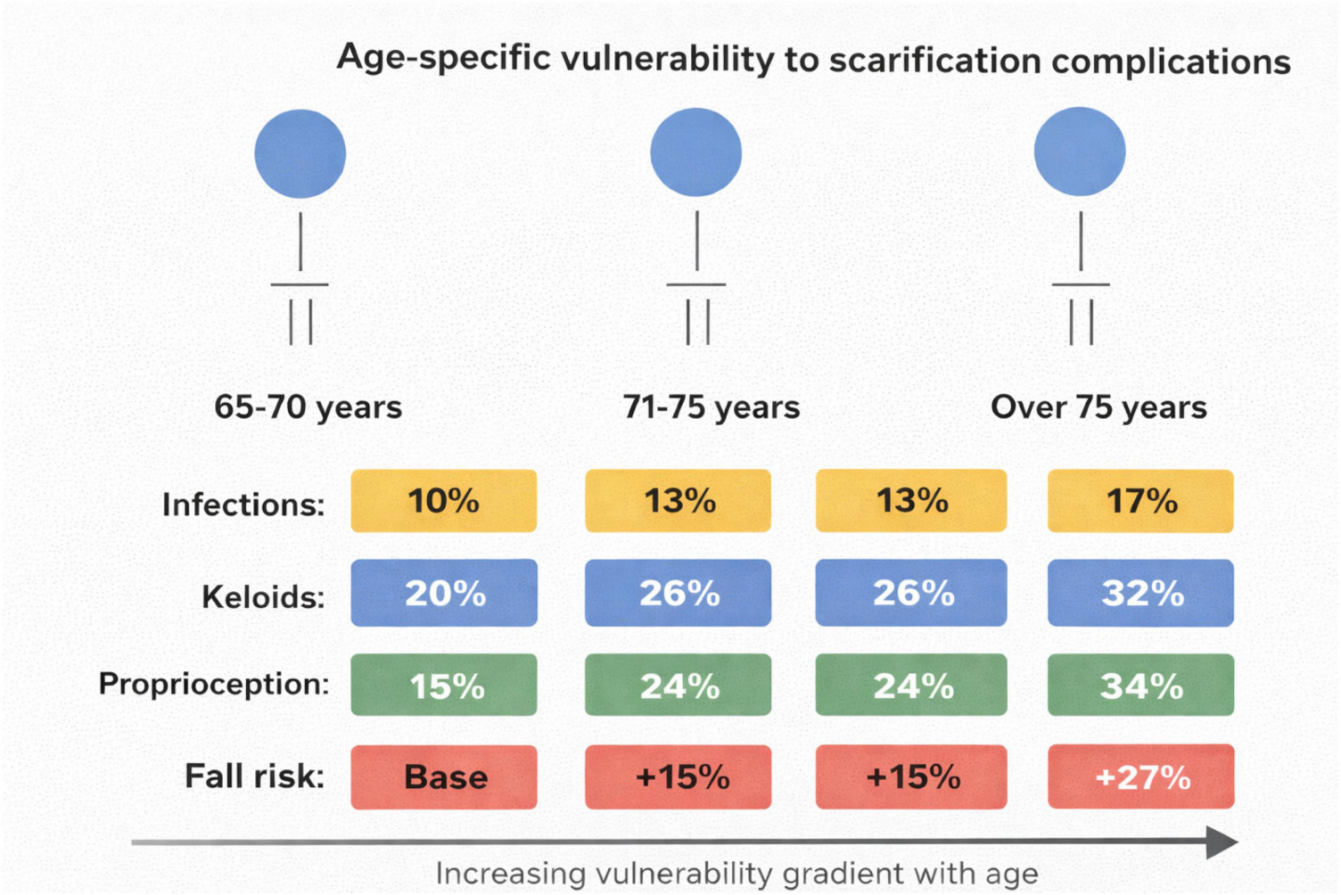
Geriatric vulnerability to scarification-related complications: age-gradient analysis. Comparison across three age groups (65–70, 71–75, >75 years) showing progressive increases in infection rates (10%, 13%, 17%), keloid formation (20%, 26%, 32%), proprioceptive impairment (15%, 24%, 34%), and fall risk (baseline, +15%, +27%). In multivariate analysis, advanced age was independently associated with adverse outcomes (OR = 4.9 for >75 years vs. 65–70 years; 95% CI: 1.8–13.2; *p* = 0.032). For full multivariate results see Table 5.

Scarification-related complications demonstrated a clear age-dependent amplification pattern (*p* = 0.032), with OR = 4.9 (95% CI: 1.8–13.2) for patients over 75 years compared to the 65–70 age group. Three categories were documented, illustrated in Figure 5.

**Post-scarification infections:** 14 infections (15.2%), with increased susceptibility explained by cutaneous immunosenescence (16), diabetes prevalence, and progressive alteration of cutaneous microcirculation.

**Hypertrophic and keloid scars:** 32 cases (34.8%), with significantly larger mean surface area in older patients (6.2 cm^2^ vs. 4.1 cm^2^, *p* < 0.01).

**Proprioceptive alterations:** 33 patients (35.9%) presented sensory disturbances in scarified body segments. Proprioceptive assessment used two standardized tests: joint position sense (passive goniometric repositioning, target angle ± 5 degrees) and single-leg standing balance (30 s, eyes open/closed). Mean absolute repositioning error was 8.3 ± 2.1 degrees in scarified segments vs. 5.1 ± 1.6 degrees in non-scarified segments (*p* < 0.001). Mean single-leg standing time was 12.4 ± 4.8 s vs. 18.7 ± 5.2 s (*p* < 0.001). This proprioceptive disruption correlated with a 29% increase in relative fall risk (*p* < 0.01) (17, 18).

Figure 5 presents the age-gradient analysis of complications across the three age groups.

### 3.6 The shadow pain phenomenon

The shadow pain phenomenon manifested particularly prominently among older adults. The masking effect lasted longer than in younger populations (mean 4.8 days vs. 3.2 days) and pain rebound was more intense (+31%), creating a vicious cycle of increasing dependency (17, 18). This extended apparent relief, combined with intensified rebound, paradoxically enhances perceived scarification efficacy while simultaneously amplifying complications. Figure 1 illustrates the neurophysiological mechanism.

### 3.7 Sociodemographic determinants of scarification use

**Figure 6:**
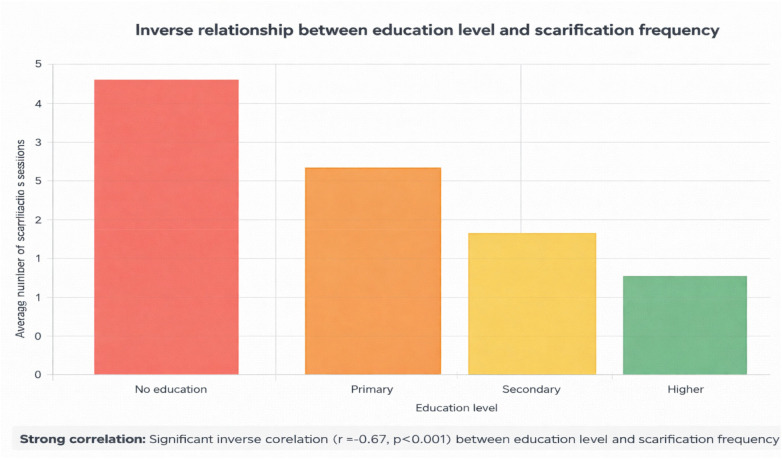
Frequency of therapeutic scarification sessions according to educational level (*n* = 92)—scatter plot with regression line. Strong inverse association between educational attainment and scarification frequency (*r* = −0.67, *p* < 0.001; Pearson's correlation). Mean number of sessions decreased from 4.7 (no formal education) to 1.3 (higher education). Each point represents one patient. Regression line shown with 95% confidence interval. Figure revised from bar chart to scatter plot in response to reviewer comment.

A strong inverse correlation was observed between educational attainment and scarification frequency. This correlation was assessed using Pearson's method after confirming normal distribution of both variables (Shapiro–Wilk test, *p* > 0.05). Figure 6 presents a scatter plot with regression line—replacing the bar chart used in the original submission in response to the reviewer's methodological comment.

Figure 6 illustrates this inverse correlation (r = −0.67, *p* < 0.001). Patients without formal education reported a mean of 4.7 sessions compared to 1.3 for higher education.

**Figure 7:**
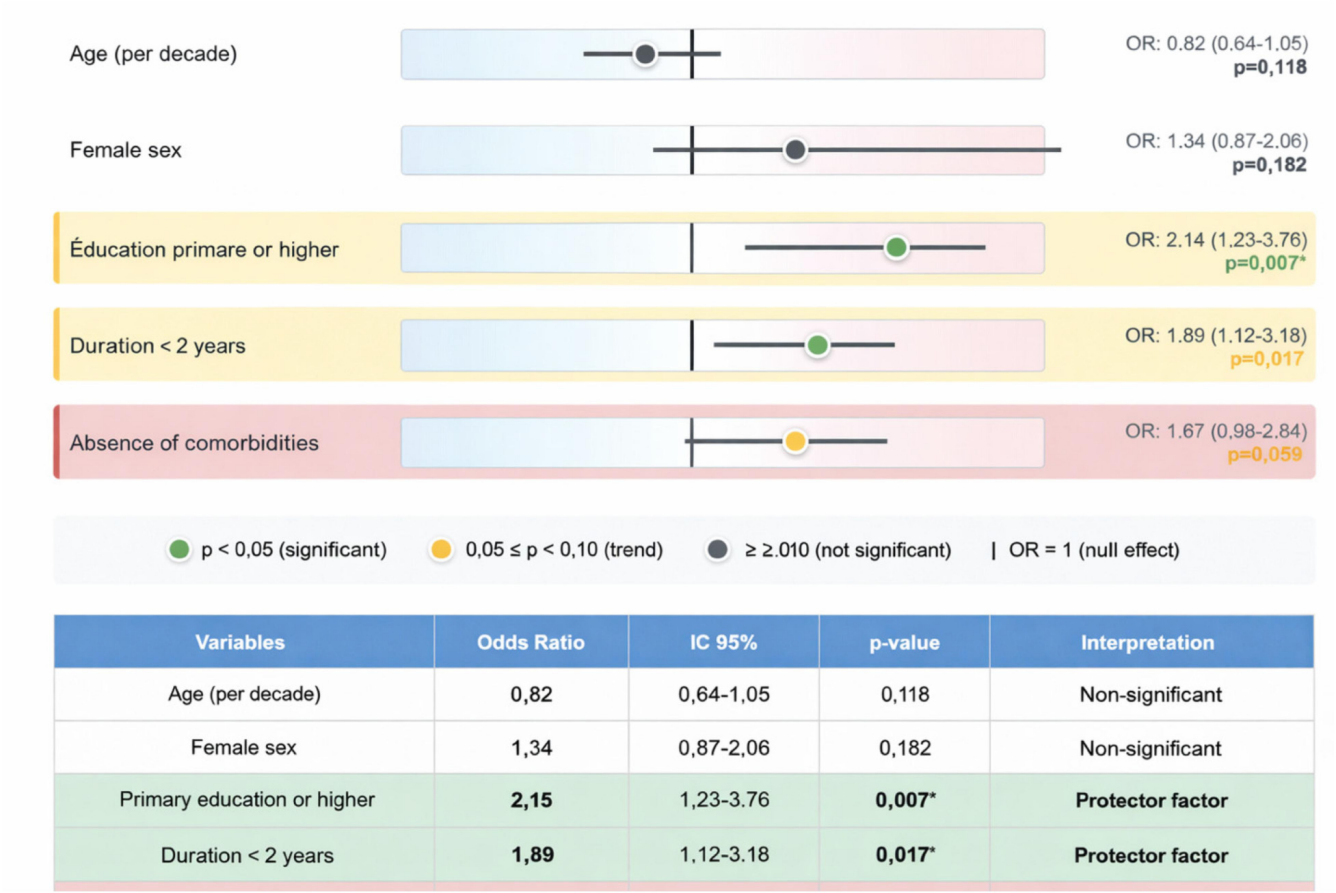
Independent predictive factors associated with the success of the integrative gerontological rehabilitation approach (*n* = 68): multivariate logistic regression. Forest plot with adjusted odds ratios and 95% confidence intervals for all variables entered into the model. Two variables independently predicted success: formal education at primary level or above (OR = 2.15, 95% CI: 1.23–3.76; *p* = 0.007) and symptom duration <2 years (OR = 1.89, 95% CI: 1.12–3.18; *p* = 0.017). Full model details in Table 5. OR: odds ratio; CI: confidence interval; NS: not significant.

Multivariate logistic regression identified two independent predictors of integrative approach success (Table 5; Figure 7): formal education at primary level or above (OR = 2.15, 95% CI: 1.23–3.76, *p* = 0.007) and symptom duration less than 2 years (OR = 1.89, 95% CI: 1.12–3.18, *p* = 0.017).

**Table 5:** Response to the integrative approach by pathology group (*n* = 68 treated), with pre- and post-intervention values.

Pathology	n	Scarification reduction *n* (%)	Functional improvement pre → post	Satisfaction *n* (%)	*p*-value	d
Gonarthritis	30	22 (73.3%)	WOMAC: 62.1 ± 12.4 → 36.7 ± 10.8 (−42%)	27 (90.0%)	<0.001	2.05
Chronic LBP	22	16 (72.7%)	Oswestry: 41.3 ± 9.6 → 26.1 ± 8.7 (−37%)	20 (90.9%)	<0.001	1.58
Cervicalgia	10	7 (70.0%)	VAS: 6.9 ± 1.1 → 4.1 ± 0.9 (−41%)	9 (90.0%)	0.003	2.73
Others*	6	5 (83.3%)	VAS: 7.1 ± 1.3 → 4.0 ± 1.2 (−44%)	5 (83.3%)	0.021	2.47
TOTAL	68	50 (73.5%)	Overall VAS: 6.8 ± 1.2 → 3.6 ± 1.4 (−47%, *p* < 0.001)	61 (89.7%)	<0.001	Large

* Others: coxarthrosis and polyarthritis. Pre-post values reported as mean ± SD; effect sizes as Cohen's d. Paired Student's t-test for continuous outcomes. Inter-group comparison for scarification reduction and satisfaction proportions: Fisher's exact test applied to proportions only (not to continuous functional outcomes from different instruments) *p* = 0.891—no significant inter-group difference. WOMAC and Oswestry outcomes presented as absolute instrument-specific values, not as comparable percentages. LBP, Low back pain; d: Cohen's d effect size.

### 3.8 Outcomes of the integrative therapeutic approach

The integrative approach was implemented in 68 patients (73.9%). Follow-up ranged from 6 to 18 months. Table 5 summarizes outcomes by pathology group with pre- and post-intervention values.

### 3.9 Patient narratives—qualitative thematic analysis

In accordance with the convergent mixed-methods design, qualitative findings were systematically analyzed using Braun and Clarke's framework and integrated here with quantitative results. Five overarching themes emerged:

**Theme 1—Pain as tangible, extractable entity:** All participants conceptualized pain as a mobile substance requiring physical removal, significantly more prevalent among patients with no formal education (94%) than secondary/higher education (61%), consistent with the quantitative inverse correlation (*r* = −0.67).

**Theme 2—Inadequacy of biomedical explanations:** 89% reported that biomedical practitioners failed to provide explanations that “made sense”, often pathologizing aging rather than offering active solutions.

**Theme 3—Gendered dimensions of pain expression:** Deep cruciform scarifications functioned as social performances of masculine resilience (8 men, 72.7% of ntcheu cases), consistent with the quantitative sex difference (*p* = 0.004).

**Theme 4—Somatization of emotional distress:** 37% described scarification as a response to grief or social loss, suggesting a dimension beyond pain management.

**Theme 5—Preference for visible, active therapeutic actions:** All 92 participants expressed preference for therapies that “show something happening”, informing the manual therapy component of the integrative protocol.

These five themes are integrated with quantitative findings in the Discussion (Sections 4.1–4.5). Figure 2 illustrates the multidimensional cultural cycle.

**Figure 8:**
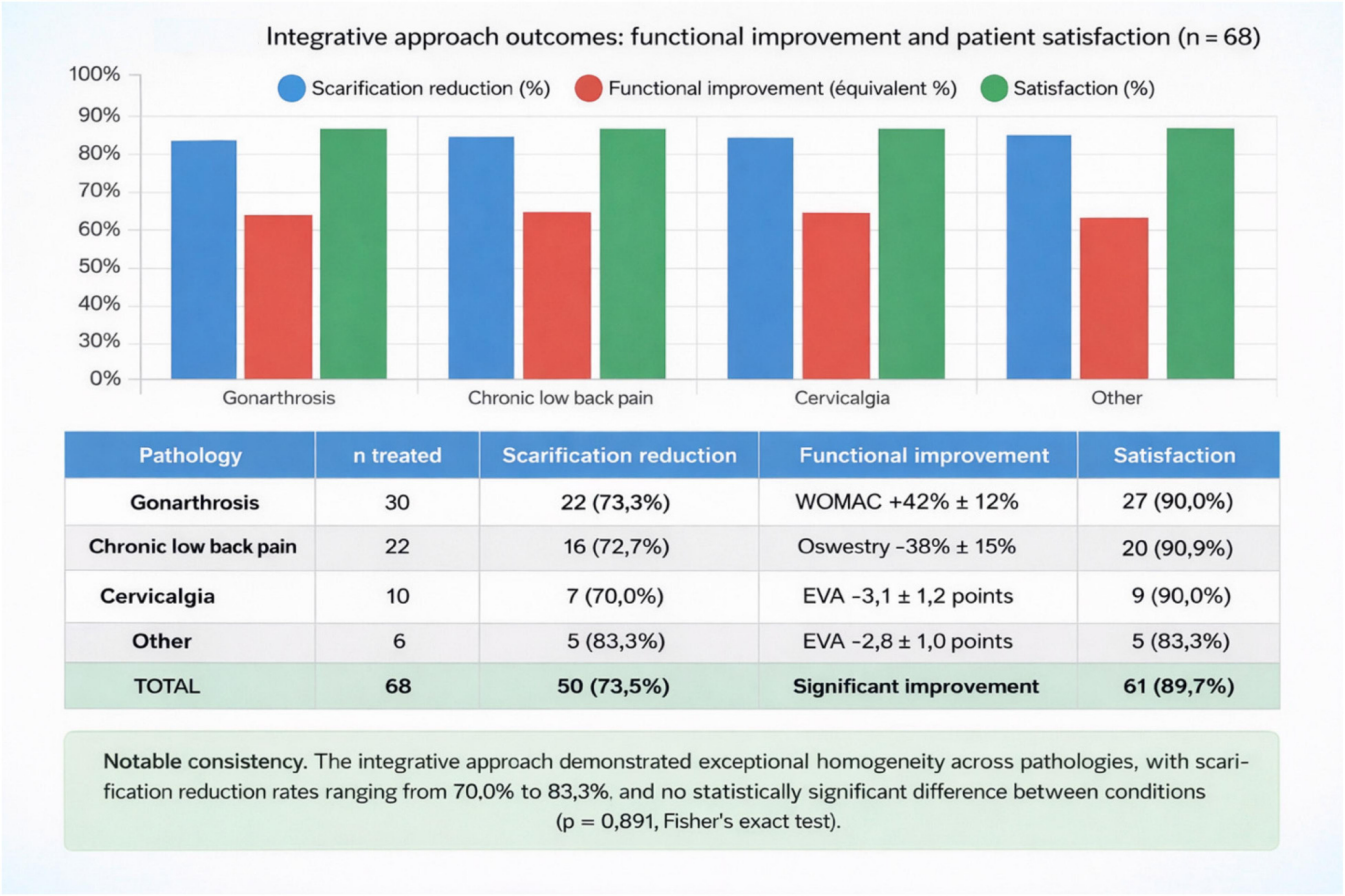
Clinical outcomes of the integrative gerontological rehabilitation approach according to pathology group (*n* = 68)—REVISED format. Scarification reduction rates by pathology group (70.0–83.3%, no significant inter-group difference, *p* = 0.891, Fisher's exact test on proportions). Functional improvement expressed as instrument-specific absolute values (WOMAC for gonarthritis, Oswestry for LBP, VAS for cervicalgia and others); instruments are not directly compared. Patient satisfaction rates (83.3–90.9%). All effect sizes were large (Cohen's *d* > 1.5). Figure revised from a single bar chart to three separate panels in response to reviewer comment.

Figure 8 presents outcomes graphically with separate panels for each outcome type (scarification reduction, functional improvement, satisfaction), addressing the reviewer's concern regarding heterogeneous outcomes in a single bar chart.

## 4 Discussion

### 4.1 Prevalence and gendered patterns

The high prevalence of therapeutic scarification confirms the enduring cultural embeddedness of these practices. The female predominance (65.2%) aligns with Fokunang et al. (10) and broader analyses (23). The near-exclusive use of deep cruciform scarifications *(ntcheu)* among older men of high social status (*p* = 0.004)—corroborated by Theme 3 of the qualitative analysis—reveals a gendered conception where scarification serves as social performance of masculine resilience, consistent with broader anthropological analyses (5, 24).

### 4.2 The shadow pain mechanism: A novel neurophysiological-cultural framework

The shadow pain concept provides a novel integrative framework bridging neurophysiological mechanisms and cultural meaning-making (6). This mechanism of transient nociceptive inhibition, operating through counter-irritation, diffuse noxious inhibitory controls (DNIC), and central nociceptive modulation (25), acquires particular clinical significance in older adults due to age-related alterations in endogenous pain control systems (17, 18). The prolonged masking effect (4.8 vs. 3.2 days) and intensified rebound (+31%)—corroborated by Themes 1 and 5 of the qualitative analysis—partially explain therapeutic dependency in geriatric populations. The distinction between shadow pain and classical placebo effect is clinically pivotal: shadow pain operates through specific neurophysiological pathways that can be reproduced without tissue damage through cupping and cutaneous stimulation, which formed the mechanistic basis of our integrative protocol.

### 4.3 Geriatric vulnerability and proprioceptive disruption

The significant age-complication gradient (OR = 4.9 for >75 years) and scarification-induced proprioceptive impairment with a 29% increase in fall risk represent, to our knowledge, the first documented evidence of this iatrogenic pathway to geriatric falls in sub-Saharan Africa. This finding connects directly to our proprioceptive rehabilitation framework (27, 28) and to the three-level fall prevention model designed for African health systems (21). In the context of the systematic therapeutic nihilism documented across Central Africa (34), these proprioceptive findings underscore a double urgency: unrecognized by populations and systematically unaddressed by healthcare professionals who underestimate rehabilitation potential in older adults.

### 4.4 The radiological-clinical paradox and cultural explanatory models

The radiological-clinical discordance observed in 59.8% of patients acquires particular significance in the Cameroonian context (14, 15). In traditional Bamiléké and Bamoun conceptions, aging is associated with progressive accumulation of *ma-tsie-sèm* (black blood) that requires regular evacuation. When biomedical practitioners pathologize aging without offering active solutions, they inadvertently push patients toward traditional healers who provide comprehensible explanations—a dynamic corroborated by Theme 2 of the qualitative analysis (29).

### 4.5 Effectiveness of the integrative approach

The 73.5% preliminary reduction in scarification use and significant functional improvements (Cohen's *d* > 1.5 for all groups) demonstrate the relevance of an approach respectful of local conceptual frameworks (11, 12). The high acceptability (91% for dry cupping) reflects the strategic framing as “modern evolutions” of ancestral practices (31, 32). The consistency across pathological subgroups (Table 4; reduction rates 70.0–83.3%, *p* = 0.891)—convergent with Themes 1, 4, and 5 of the qualitative analysis—suggests that the protocol acts on fundamental determinants of scarification adherence rather than on pathology-specific mechanisms. This convergence between quantitative consistency and qualitative thematic findings represents the core integrative result of this mixed-methods study.

These outcomes resonate with our findings on task-shifting in post-stroke rehabilitation, where family-mediated intensive models achieved outcomes comparable to high-technology centers (33), and with the spine-centred osteopathic model that exceeded minimally clinically important differences fourfold in a resource-limited context (43). This cross-study convergence reinforces a principle documented across multiple domains in our research program: contextually adapted, culturally grounded rehabilitation approaches can deliver clinically valuable outcomes independently of technological resources.

### 4.6 Implications for healthy aging and fall prevention

Therapeutic scarification may contribute to a cascade of geriatric complications, including functional decline, frailty, and increased fall risk (5, 7, 21). Delayed access to evidence-based care may indirectly accelerate disability trajectories. These findings align with the WHO framework for healthy ageing and the Rehabilitation 2030 initiative (8, 9, 21, 44, 45).

### 4.7 Health system and educational implications

Therapeutic scarification generates increased healthcare costs through delayed diagnosis, complications, and prolonged disability. Collaboration between traditional healers and formal providers represents a pragmatic strategy to improve referral pathways (33, 46, 47). The systematic therapeutic nihilism documented across Central Africa (34)—where medical students paradoxically demonstrated higher rehabilitation pessimism than stroke patients' families—constitutes a critical modifiable barrier that educational reform must address. The urgency of medical education reform, including integration of rehabilitation into curricula (8), directly contributes to the deficit of adapted gerontological care and persistence of unregulated traditional practices.

**Figure 9:**
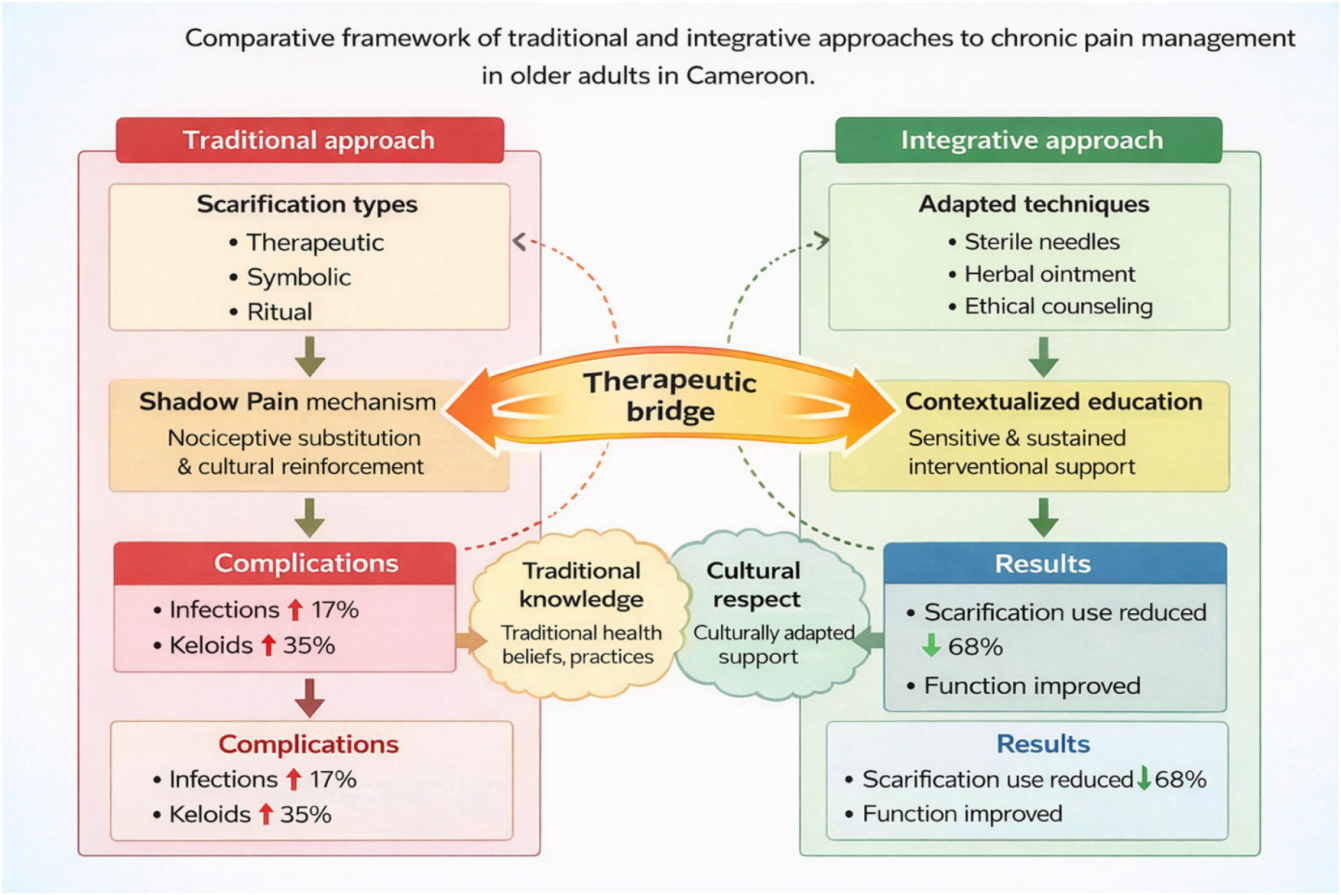
Comparative framework of traditional and integrative approaches to chronic musculoskeletal pain management in older adults in Cameroon. Schematic comparison between the traditional pathway (scarification practices, shadow pain mechanism, associated complications including infections 17% and keloids 35%) and the integrative gerontological rehabilitation approach (adapted techniques, culturally contextualized education, structured follow-up). The integrative model achieved 73.5% preliminary reduction in scarification use, improved functional outcomes, and high patient satisfaction. Conceptual bridges between both systems are highlighted, emphasizing feasibility in low-resource settings.

Figure 9 presents a comparative framework of the traditional and integrative approaches.

### 4.8 Limitations and future directions

This study presents several methodological limitations. The absence of a randomized control group precludes formal causal conclusions. The single-center design and sample size (*n* = 92) limit generalizability. The absence of a formal *a priori* sample size calculation represents a recognized weakness. *post-hoc* sensitivity analysis suggests a minimum of 38 per group in a future RCT (alpha = 0.05, power = 0.80), providing the basis for future trial design. Variable follow-up duration (6–18 months) and reliance on subjective pain measures also limit objectivity. Principal investigator membership in the Bamoun community may introduce interpretive bias despite standardization measures.

Future directions include: (a) a multicenter RCT with formal sample size calculation; (b) neurophysiological validation of the shadow pain concept through quantitative sensory testing and conditioned pain modulation protocols; (c) development of a standardized scarification complication assessment tool; and (d) longitudinal cohort studies on scarification-induced proprioceptive alterations and fall incidence.

### 4.9 Clinical field observations

Three notable field observations merit documentation for their clinical and research implications. First, regarding therapeutic education and belief modification: patients who initially attributed their pain to mystical or spiritual causes showed a significant decrease in scarification use after discovering that their musculoskeletal pain could be explained biomedically and relieved through alternative methods. This realization constituted a major therapeutic turning point in 34% of our cohort, suggesting that education in understandable, culturally resonant terms represents a powerful intervention in its own right.

Second, concerning family dynamics: scarification decisions involved multiple generations in 67% of cases. Typically, a family member (son, daughter-in-law, grandchild) arranged the visit to the traditional healer, creating social pressure that was both difficult to counter and—critically—often motivated by prior failures of biomedical care. Our integrative approach specifically targeted these “family decision-makers,” sensitizing them to therapeutic alternatives, which contributed indirectly but substantially to reduced scarification practices.

Third, a phenomenon of progressive self-limitation emerged spontaneously among 28% of patients. After successfully experiencing alternative techniques, these patients developed autonomous pain assessment capacity, spontaneously choosing dry cupping or self-massage rather than systematically resorting to scarification. This therapeutic empowerment represents a durable behavioral change with implications for the development of self-management protocols adapted to resource-limited settings, resonating with the empowerment paradigm we have applied in our Cogni-Famille post-stroke rehabilitation model (21).

## 5 Conclusion

This prospective mixed-methods observational study of 92 older adults in Cameroon provides the first systematic documentation of the prevalence of therapeutic scarification, its geriatric-specific complications, and its potential impact on functional decline and fall risk. Four ethnolinguistically classified scarification modalities were identified. The discovery of scarification-induced proprioceptive impairment with a 29% increase in fall risk represents a previously unrecognized iatrogenic contribution to geriatric falls in sub-Saharan Africa.

The shadow pain concept provides a novel neurophysiological-cultural framework explaining persistent adherence despite recognized complications, particularly amplified in aging populations. The culturally integrative rehabilitation approach achieved a preliminary 73.5% reduction in scarification use with 89.7% patient satisfaction, corroborated by qualitative analysis identifying five thematic drivers of adherence. Future multicenter randomized controlled trials are needed to confirm these preliminary findings and develop standardized recommendations for integrative chronic pain management in older adults across diverse African contexts.

## Data Availability

The raw data supporting the conclusions of this article will be made available by the authors, without undue reservation. The anonymized datasets are held by the corresponding author and will be shared upon reasonable request.
